# Molecular basis of diseases induced by the mitochondrial DNA mutation m.9032T>C

**DOI:** 10.1093/hmg/ddac292

**Published:** 2022-11-26

**Authors:** Emilia Baranowska, Katarzyna Niedzwiecka, Chiranjit Panja, Camille Charles, Alain Dautant, Jean-Paul di Rago, Déborah Tribouillard-Tanvier, Roza Kucharczyk

**Affiliations:** Institute of Biochemistry and Biophysics, Polish Academy of Sciences, 02-206 Warsaw, Poland; Institute of Biochemistry and Biophysics, Polish Academy of Sciences, 02-206 Warsaw, Poland; Institute of Biochemistry and Biophysics, Polish Academy of Sciences, 02-206 Warsaw, Poland; Univ. Bordeaux, CNRS, IBGC, UMR 5095, F-33000 Bordeaux, France; Univ. Bordeaux, CNRS, IBGC, UMR 5095, F-33000 Bordeaux, France; Univ. Bordeaux, CNRS, IBGC, UMR 5095, F-33000 Bordeaux, France; Univ. Bordeaux, CNRS, IBGC, UMR 5095, F-33000 Bordeaux, France; Institute of Biochemistry and Biophysics, Polish Academy of Sciences, 02-206 Warsaw, Poland

## Abstract

The mitochondrial DNA mutation m.9032T>C was previously identified in patients presenting with NARP (Neuropathy Ataxia Retinitis Pigmentosa). Their clinical features had a maternal transmission and patient’s cells showed a reduced oxidative phosphorylation capacity, elevated reactive oxygen species (ROS) production and hyperpolarization of the mitochondrial inner membrane, providing evidence that m.9032T>C is truly pathogenic. This mutation leads to replacement of a highly conserved leucine residue with proline at position 169 of ATP synthase subunit *a* (L_169_P). This protein and a ring of identical *c*-subunits (*c*-ring) move protons through the mitochondrial inner membrane coupled to ATP synthesis. We herein investigated the consequences of m.9032T>C on ATP synthase in a strain of *Saccharomyces cerevisiae* with an equivalent mutation (L_186_P). The mutant enzyme assembled correctly but was mostly inactive as evidenced by a  > 95% drop in the rate of mitochondrial ATP synthesis and absence of significant ATP-driven proton pumping across the mitochondrial membrane. Intragenic suppressors selected from L_186_P yeast restoring ATP synthase function to varying degrees (30–70%) were identified at the original mutation site (L_186_S) or in another position of the subunit *a* (H_114_Q, I_118_T). In light of atomic structures of yeast ATP synthase recently described, we conclude from these results that m.9032T>C disrupts proton conduction between the external side of the membrane and the *c*-ring, and that H_114_Q and I_118_T enable protons to access the *c*-ring through a modified pathway.

## Introduction

Oxidative phosphorylation (OXPHOS) provides eukaryotic cells with the energy-rich ATP molecule (1). During this process, electrons from carbohydrates and fatty acids are transferred to oxygen, which results in a proton gradient across the mitochondrial inner membrane that is used by the ATP synthase to phosphorylate ADP with inorganic phosphate. Mutations that compromise this activity result in devastating neuromuscular diseases (2–4). Many have been located in the mitochondrial genome where are the genes of 13 OXPHOS proteins and of a number of transfer and ribosomal RNAs that are required for their synthesis inside the mitochondrion (5). With the advent in recent years of high-resolution structures of these proteins and a detailed description of their energy-transducing mechanisms, it has become possible to make predictions about the possible consequences on mitochondrial function of specific mutations in their genes. However, only a limited number of amino acid residues in OXPHOS proteins have known critical function and these are generally not the target of the mutations found in patient’s mitochondrial DNA. Furthermore, these mutations usually affect only a fraction of the numerous copies of the mitochondrial genome (heteroplasmy) and many other sources of genetic heterogeneity in nuclear and mitochondrial DNA exist between individuals, which makes it difficult to evaluate their functional consequences and pathogenicity. Last but not least, there are still no reliable methods for genetically transforming human mitochondria.

Due to these difficulties, the yeast *Saccharomyces cerevisiae* has been used as a model system for evaluating the functional consequences of mtDNA mutations found in patients (6–10). Its mitochondrial genome can be manipulated (11) and owing to the strong instability of heteroplasmy in this organism (12), strains homoplasmic for a specific mutation of this DNA can be obtained quite easily. Importantly also, the structures of the mtDNA encoded proteins have been highly conserved from yeast to humans (13). Therefore, discrete alterations in these structures should have similar consequences on mitochondrial function in evolutionary distant mitochondria. Consistently, equivalents of human mtDNA mutations leading to severe clinical phenotypes proved to compromise much more severely oxidative phosphorylation in yeast than mutations resulting in milder health problems (6,14).

We herein investigate the consequences in yeast of a mitochondrial DNA mutation (m.9032T>C) recently detected in patients presenting with the NARP syndrome (15–17). The disease was inherited maternally; its severity correlated with the level of heteroplasmy and patient’s cells showed a diminished oxidative phosphorylation capacity, leaving no doubt that this mutation is pathogenic. The m.9032T>C mutation is located in the gene *ATP6* that encodes the subunit *a* of ATP synthase (18). Together with a ring of identical *c* subunits, the subunit *a* moves protons through the membrane domain (F_O_) of ATP synthase, which is coupled to ATP synthesis in its extra-membrane domain (F_1_). As it leads to replacement of a highly conserved leucine residue with proline at position 169 of the human subunit *a* (L_169_P), in close proximity to other well conserved residues (see below), it made sense to investigate its consequences in a yeast strain with an equivalent mutation in subunit *a* (L_186_P). Based on the results herein reported, and in light of atomic structures of ATP synthase recently described (13,19–22), we propose a molecular mechanism by which m.9032T>C compromises ATP synthase function and human health.

## Results

### Yeast cells with an equivalent of the m.9032T> C mutation (L_186_P) do not grow using respiratory carbon sources and have a relatively high propensity to lose the mitochondrial genome

**Figure 1:**
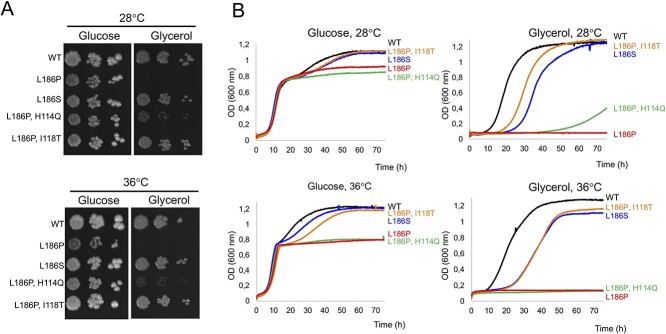
Influence of the subunit *a* mutations on the growth of yeast. (**A**) Fresh glucose cultures of the subunit *a* mutants and *WT* yeast were serially diluted and each dilution spotted on rich glucose and glycerol plates. The plates were photographed after three days of incubation at the indicated temperature. (**B**) Growth curves in liquid glucose and glycerol media. The shown data are representative of three independent experiments.

The m.9032 T > C mutation results in the substitution of a highly conserved leucine residue with proline at position 169 of the human subunit *a*, 186 in the yeast mature protein (196 in the precursor form of yeast subunit *a* of which the first ten residues are cleaved during assembly (23)) (see below). Two nucleotide changes were introduced to replace the leucine codon 196 of the yeast *ATP6* gene with a proline codon (TTA_196_CCA). The influence of the L_186_P mutation on the growth of yeast was investigated on solid and in liquid media, from fermentable (glucose) and respiratory (glycerol) carbon sources, at 28°C and 36°C (Fig. 1). While the mutant was as expected able to grow from glucose it totally failed to multiply from glycerol at both temperatures, indicating a very severe impairment of ATP synthase function. In the shown glucose growth curves (Fig. 1B), the respiratory deficiency of L_186_P yeast was apparent once the glucose present in the media had been entirely converted into ethanol, the metabolism of which requires the presence of functional mitochondria.

**Figure 2:**
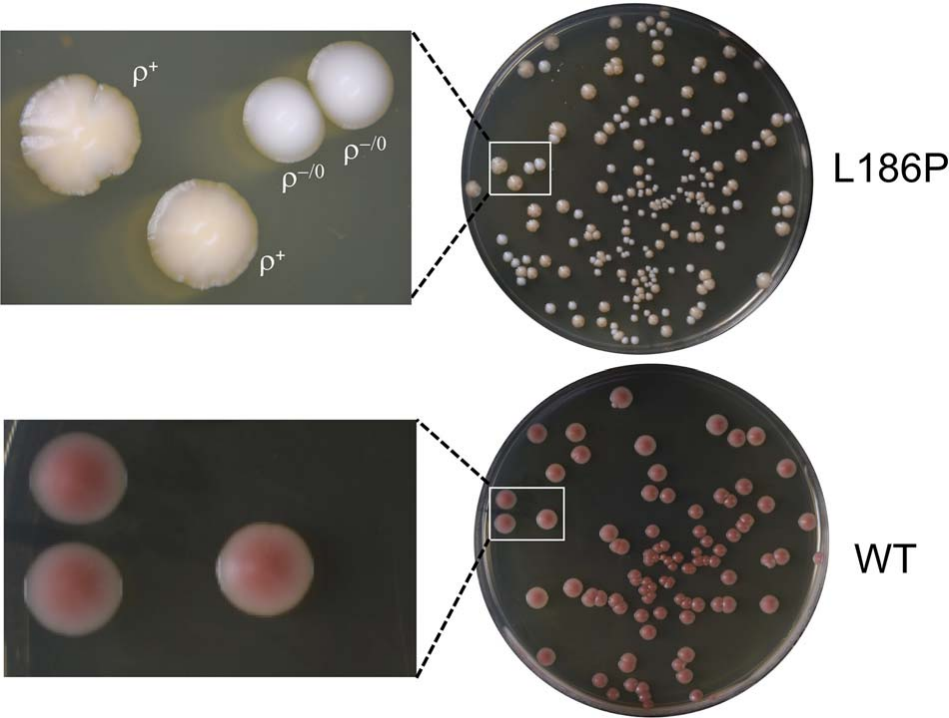
Influence of the L_186_P mutation on the stability of mitochondrial DNA. Samples from glucose cultures and L_186_P and WT yeasts were plated from single colonies on rich glucose plates and photographed after one-week incubation (see text for details).

Yeast strains with severe defects in ATP synthase have a relatively high propensity to produce cells with large deletions (ρ^−^) or total absence (ρ^0^) of mitochondrial DNA, up to 100% *vs* 5–10% in *WT* ( (24–27), see below). We therefore probed the mitotic stability of L_186_P yeast. To this end, samples of glucose cultures of L_186_P and *WT* yeasts were plated for single colonies on rich glucose plates. As expected, due to the presence of the *ade2* mutation in these strains, the colonies from *WT* yeast were red whereas those from the L_186_P mutant were much less colored (the red color does not develop well with respiratory deficient cells (28)) (Fig. 2). An important fraction (40–50%) of the colonies from L_186_P yeast were totally white and had a regular contour, indicating that they originated from ρ^−^/ρ^0^ cells. The remaining ones had a cream color and were scalloped indicating that they originated from genetically instable ρ^+^ cells. The presence/absence of ρ^+^ mtDNA in the colonies produced by L_186_P yeast was confirmed by crossing with SDC30, as described in Materials and Methods. Based on these tests, glucose cultures of the L_186_P yeast were estimated to contain about 50–60% ρ−/ρ^0^ cells.

### Intragenic suppressors of L_186_P

Taking advantage of its failure to sustain grow from respiratory carbon sources, we searched for genetic suppressors of the L_186_P mutation restoring at least partially respiratory growth and hence ATP synthase function, an approach we already used to better understand the deleterious mechanisms of a number of subunit *a* mutations (29–33). To this end, glucose-grown L_186_P cells were plated as dense layers on solid glycerol medium (10^8^ cells/plate). Twelve respiring clones that emerged from the glycerol plates after several days of incubation were analyzed by DNA sequencing for the presence of novel mutations in the *ATP6* gene (intragenic suppressors). Three different mutations were identified: a first-site reversion leading to replacement of the mutant proline residue with serine (referred to as L_186_S to indicate the amino acid change relative to the wild type protein) in four clones, and two second-site reversions in another position of the *ATP6* gene protein: H_114_Q (in four clones) and I_118_T (in four clones) (see Table 1 for the corresponding nucleotide changes). The L_186_S and L_186_P, I_118_T strains showed fast growth in glycerol, while the L_186_P, H_114_Q mutant grew much less rapidly (Fig. 1). The fast growth of L_186_S and L_186_P, I_118_T strains (in the exponential phase) does not mean that ATP synthase function is unaffected. Previous studies have indeed shown that large ATP synthase activity deficits (of at least 80%) are needed to affect obviously the growth rate of yeast in respiratory conditions (34,35). Consistent with the restoration of respiratory growth, the three revertant strains showed a much better capacity than L_186_P yeast to maintain the mitochondrial genome with only <10% ρ^−^/ρ° cells (*vs* 50–70% for the original mutant) (Table 2).

**Table 1:** Mutations in yeast subunit *a*

Codon change	Amino acid change
Original mutation	
TTA_196_CCA	L_186_P
Intragenic suppressors	
TTA_196_TCA	L_186_S
TTA_196_CCA + CAT_124_CAA	L_186_P, H_114_Q
TTA_196_CCA + ATT_128_ACT	L_186_P, I_118_T

Amino acid numbers are those in yeast mature subunit *a* without the first ten residues present in its precursor form.

Each suppressor was identified in four genetically independent isolates.

### Assembly/stability of ATP synthase

**Figure 3:**
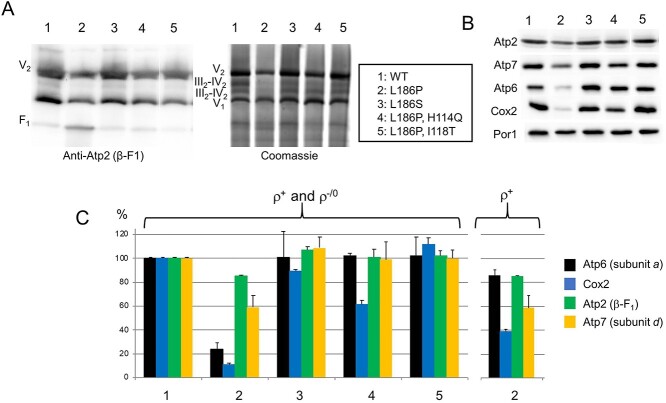
Influence of the subunit *a* mutations on the assembly/stability and abundance of ATP synthase and Complex IV. (**A**) Mitochondria isolated from the subunit *a* mutants and *WT* yeast grown in rich galactose at 28°C were solubilized with digitonin (1.5 g/g protein) and separated in a 3–12% gradient polyacrylamide BN gel (200 μg of proteins/lane). The proteins were transferred to a PVDF membrane and probed with antibodies against the Atp2 (β-F_1_) subunit of ATP synthase. The immunological signals corresponding to dimers (V_2_) and monomers (V_1_) of ATP synthase and free F_1_ particles are indicated. (**B**) Total cellular protein extracts were separated by SDS-PAGE and then transferred to a nitrocellulose membrane and probed with antibodies against the indicated proteins. (**C**) The intensities of the bands in Panel B were calculated using ImageJ, normalized to porin and expressed in % of *WT*. Because of the large amount (72%) of ρ^−^/ρ^0^ cells in cultures of the L_186_P mutant (where the subunit *a* and Cox2 cannot be synthesized), the levels of these two proteins were calculated for the part of the population (28%) that contained complete (ρ^+^) mtDNA (shown on the *right*). The standard errors were calculated from three independent experiments.

The influence of the subunit *a* mutations on the assembly/stability of ATP synthase was analyzed by BN- and SDS-PAGE of mitochondrial extracts prepared from cells grown in rich galactose medium. Fully assembled F_1_F_O_ dimers and monomers and free F_1_ particles were detected in BN gels for all the mutants as in *WT* yeast, using antibodies against the β (Atp2) subunit of F_1_ (Fig. 3A). Free F_1_ was more abundant in samples from the L_186_P mutant *vs* the other strains, which reflects its strong propensity to produce ρ^−^ρ^0^ cells. These cannot synthetize the three mtDNA-encoded subunits of F_O_ (Atp6/*a*, Atp8 and Atp9/*c*) whereas the F_1_ is entirely encoded by nuclear genes and can assemble in the absence of F_O_ (36). Quantitative estimation of ATP synthase was performed by measuring the levels of the subunit *a* in denaturing gels. Owing to its high susceptibility to degradation when not assembled, this subunit is a good indicator of fully assembled ATP synthase. The levels of subunit *a* were almost the same in the analyzed strains except in L_186_P yeast where they were decreased by about 70–80% *vs WT* (Fig. 3B and C). The low abundance of the subunit *a* in the L_186_P mutant mostly results from its high propensity to produce ρ^−^/ρ^0^ cells rather than a compromised ability of the mutant protein to assemble. Indeed, when expressed relative to the amounts of ρ^+^ cells in the cultures used for these experiments, a good accumulation of subunit *a* was estimated in the L_186_P mutant (84% *vs WT*, see Fig. 2C). Thus, the V_1_ and V_2_ immunological signals in the shown gels correspond to fully assembled ATP synthase only, in line with previous studies showing that incomplete ATP synthase assemblies lacking subunit *a* are fragile and easily dissociate in BN-gels (26,37).

### Mitochondrial respiration and ATP synthesis

We next evaluated the influence of the subunit *a* mutations on oxidative phosphorylation by measuring the rates of electron transfer to oxygen and ATP synthesis in intact (osmotically protected) mitochondria using NADH as a respiratory substrate. These activities were very weak in L_186_P mitochondria (<10% *vs WT*), whereas those from the revertant strains respired and produced ATP more rapidly albeit not as fast as *WT* mitochondria (Table 2). The yield in ATP per electron transferred to oxygen (P/O) in mitochondria from the L_186_P, I_118_T and L_186_S strains was quite normal whereas it was about half reduced in those from the L_186_P, H_114_Q strain indicating that only part of the protons that enters the F_O_ in this latter strain is properly vehiculated by the *c*-ring motor of ATP synthase and coupled to ATP synthesis in the F_1_ catalytic domain (see below). The P/O value was also strongly decreased in mitochondria from the L_186_P mutant but because of their extremely low electron transfer activity and blockade of the F_O_, a large part of the protons pumped by the respiratory chain is certainly passively returned to the mitochondrial matrix through the phospholipid bilayer of the inner membrane, thus without any ATP synthesis.

No significant difference was observed between the rates of respiration measured in absence and presence of oligomycin in the analyzed strains (the values measured in the presence of the drug are not shown), indicating that none of the mutations led to important passive proton leaks through the F_O_ (like those observed in strains with mutations in the central stalk subunits of ATP synthase (38–40)). Such leaks have thus far never been observed in yeast subunit *a* mutants and the ability of mitochondria from the L_186_P mutant to sustain a significant and stable electrochemical potential across the inner membrane (see below) argues against the existence of such leaks in this mutant.

**Table 2:** Mitochondrial respiration and ATP synthesis/hydrolysis activities

Strain	Respiration rates (nmol O.min^−1^.mg^−1^)				ATP synthesis rate (nmol Pi.min^−1^.mg^−1^)		P/O	ρ^0/−^ (%)	ATPase activity (μmol Pi.min^−1^.mg^−1^)	
	NADH	NADH +ADP	NADH +CCCP	Asc/TMPD + CCCP	− oligo	+ oligo			− oligo	+oligo
WT	732 ± 68	1413 ± 50	2367 ± 163	4086 ± 102	1886 ± 3	132 ± 12	1.29 ± 0.02	1 ± 1	2.834 ± 0.010	0.261 ± 0.20
L_186_P	78 ± 38	93 ± 13	106 ± 40	301 ± 1	38 ± 1	13 ± 0.3	0.41 ± 0.01	72 ± 9	0.605 ± 0.038	0.508 ± 0.034
L_186_S	456 ± 55	953 ± 93	2044 ± 124	3659^*^ ± 126	1155 ± 131	50 ± 54	1.19 ± 0.02	1 ± 1	1.798 ± 0.290	0.174 ± 0.044
L_186_P, H_114_Q	413 ± 15	589 ± 4	1249 ± 139	2604^*^^*^ ± 61	429 ± 61	50 ± 25	0.67 ± 0.12	8 ± 3	1.003 ± 0.439	0.269 ± 0.101
L_186_P, I_118_T	440 ± 4	926 ± 19	2092 ± 75	3485^*^ ± 56	1424 ± 25	149 ± 39	1.38 ± 0.01	6 ± 1	2.008 ± 0.167	0.292 ± 0.105

Mitochondria were isolated from cells grown for 5–6 generations in rich galactose medium (YPGalA) at 28 °C. Reaction mixes for measuring the rates of oxygen consumption and ATP synthesis contained 0.15 mg/mL proteins (osmotically-protected mitochondria at pH 6.8), 4 mM NADH, 150 μM and 1 mM ADP (in respiration and ATP synthesis assays respectively), 12.5 mM ascorbate, 1.4 mM N,N,N,N-tetramethyl-p-phenylenediamine (Asc/TMPD), 4 μM CCCP and 4 μg/mL oligomycin (oligo). The ATPase assays were performed from mitochondrial samples that had been frozen at −80 °C in the absence of osmotic protection at pH 8.4 in presence of 1 mM ATP.

Another line of evidence indicating that the L_186_P mutation prevents F_O_-mediated transport was provided by probing the levels of Complex IV’s content and activity. Previous work has shown that the rate of Complex IV biogenesis in yeast is influenced by the proton transport activity of F_O_, possibly as a way to co-regulate in cells their needs in ATP and respiration (41,42). Strains with passive proton leaks (i.e. not coupled to ATP synthesis), for instance mutants with defaults in the central stalk (38–40), keep a good capacity to assemble the Complex IV, showing that it is well the proton flow through the F_O_ rather than the rate of F_1_-mediated ATP synthesis that controls the biogenesis of Complex IV. In BN gels stained with Coomassie blue, the levels of Complex IV associated to Complex III (III_2_-IV_2_ and III_2_-IV_1_) were dramatically reduced in mitochondrial samples from L_186_P vs *WT* yeasts whereas these assemblies were much less affected in the revertant strains (Fig. 3A). These observations were corroborated by Complex IV activity measurements (Table 2) and by probing the levels of the Cox2 subunit of Complex IV in denaturing gels (Fig. 3B,C). It is interesting to note that the extent to which Complex IV’s content and activity are reduced in mitochondria from L_186_P, H_114_Q yeast (65% *vs WT*) is much less important than the drop in the rate of ATP synthesis (20% *vs WT*), which further indicates there is still in this mutant a quite good flow of protons through the F_O_ despite its poor capacity to synthesize ATP (see below).

### Mitochondrial membrane potential

**Figure 4:**
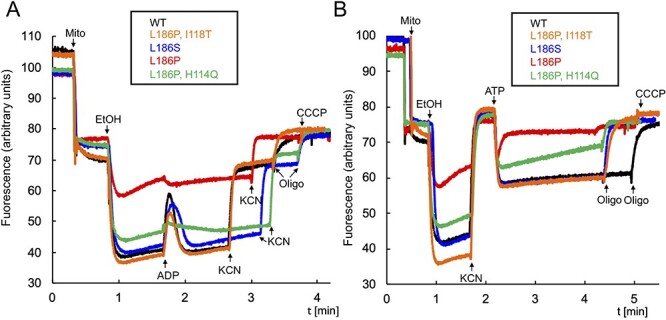
Influence of the subunit *a* mutations on mitochondrial membrane electrical potential. Variations in mitochondrial transmembrane potential (ΔΨ) were monitored by fluorescence quenching of Rhodamine 123 in intact mitochondria isolated from the subunit *a* mutants and *WT* yeast grown in rich galactose at 28°C. (**A**) ADP-driven ΔΨ consumption. (**B**) ATP-driven proton pumping. The additions were 25 μg/mL Rhodamine 123, 150 μg/mL mitochondrial proteins, 10 μL ethanol (EtOH), 75 μM ADP (2), 2 mM KCN, 4 μg/mL oligomycin (4), 4 μM CCCP (5) and 0.2 mM ATP (6). The shown traces are representative of at least three independent experiments.

We further investigated the consequences of the subunit *a* mutations by monitoring variations in transmembrane electrical potential (ΔΨ) using the cationic dye Rhodamine 123, in osmotically protected mitochondria buffered at physiological pH 6.8. As expected, adding ADP to *WT* mitochondria respiring from ethanol resulted in a sharp and transient fluorescence increase reflecting ΔΨ consumption by ATP synthase until complete phosphorylation of the added ADP (Fig. 4A). Ethanol induced a small ΔΨ in the L_186_P mitochondria and there was no significant modification in fluorescence after adding ADP. The mitochondria from the L_186_S and L_186_P, I_118_T responded quite well to ethanol and ADP whereas those from the L_186_P, H_114_Q strain took a much longer time after the addition of ADP to recover the ethanol-induced ΔΨ. A further addition of KCN to inhibit the respiratory chain resulted in only a partial ΔΨ loss in mitochondria from the *WT* and the revertant strains, and the residual ΔΨ was oligomycin-sensitive (Fig. 4A) whereas the membrane potential totally collapsed after the addition of KCN in L_186_P mitochondria. These observations are fully consistent with the measurements of oxygen consumption of ATP synthesis reported in Table 2.

In a second series of experiments, we evaluated the proton-pumping activity of ATP synthase from externally added ATP (Fig. 4B). Prior to adding ATP, the mitochondrial respiratory chain was supplied with electrons from ethanol and then inhibited with KCN, which promotes removal from the F_1_ of its natural IF1 inhibitory peptide (43). As expected adding next ATP to *WT* mitochondria results in a large and stable oligomycin-sensitive ΔΨ, reflecting F_O_-mediated proton pumping coupled to F_1_-mediated ATP hydrolysis (Fig. 4B). No ATP-driven proton pumping was detected in mitochondria from the L_186_P mutant, whereas those from the L_186_P, I_118_T and L_186_S strains responded well to ATP. A significant but weaker and less stable potential was observed with the mitochondria from the strain L_186_P, H_114_Q, which further illustrates the poor suppressor activity of the H_114_Q change compared to the two other suppressor mutations.

### Mitochondrial ATP hydrolysis

The reverse functioning of ATP synthase was further investigated by measuring the rate of ATP hydrolysis in non-osmotically protected mitochondria. In these conditions, the enzyme is not working against a proton gradient and can therefore hydrolyze ATP at its maximum rate. When F_1_ and F_O_ are properly coupled, inhibition of F_O_ with oligomycin prevents F_1_-mediated ATP hydrolysis because then the ATP synthase motor (F_1_ central stalk and *c*-ring) cannot rotate and the catalytic sites in F_1_ cannot process ATP. When their coupling is compromised, the F_1_ can hydrolyze ATP in the presence of oligomycin, for instance in ρ^−^/ρ^0^ cells unable to synthesize the F_O_ or with mutations that allow the protons to cross the F_O_ without being vehiculated by the *c*-ring. Most (90%) of the ATPase activity in *WT* mitochondria was inhibited by oligomycin and thus mediated by ATP synthase (the remaining 10% oligomycin-insensitive activity is due to other ATPases present in mitochondria, see Table 2). The ATPase activity in L_186_P mitochondria was only 20% *vs WT* and only 4% of it was inhibited with oligomycin. The strong propensity of the L_186_P mutant to produce ρ^−^/ρ^0^ cells is certainly in large part responsible for the poor inhibition by oligomycin. This cannot however explain its very poor F_1_-mediated ATPase activity since as described above ρ^+^ L_186_P cells do assemble properly the F_O_. It can be inferred that fully assembled F_1_F_O_ complexes with the L_186_P mutation have a very poor capacity to hydrolyze ATP, which further reflects the dramatic consequences of this mutation on F_O_-mediated proton transport. Mitochondrial ATPase activity was largely recovered with the L_186_S and I_118_T suppressors (65–70% *vs WT*) and efficiently inhibited with oligomycin (90%) (Table 2). In mitochondrial samples with the H_114_Q suppressor, the ATPase activity was less well restored (35% *vs WT*) and largely inhibited (75%) by oligomycin. These results perfectly mirror the ATP synthesis rate measurements, indicating that the influence of the mutations on the functioning of ATP synthase is the same whether it synthesizes or hydrolyzes ATP. The less efficient inhibition of the mitochondrial ATPase activity in the strain with the H_114_Q suppressor further supports that this mutation partially compromises the coupling of F_1_ to F_O,_ whereas there is no such energy dissipation with the two other suppressors.

## Discussion

Although the pathogenicity in humans of the L_169_P change in subunit *a* induced by m.9032T>C has been established (15–17), it was thus far not known how and to which extent it compromises ATP synthase function. This issue has been investigated in the present study using a yeast strain with an equivalent mutation in subunit *a* (L_186_P). This mutant totally failed to grow on non-fermentable substrates, providing a first indication that the L_186_P change had dramatic consequences on the ATP synthase. Consistently, although it properly assembled the mutant ATP synthase was mostly inactive as evidenced by a > 95% drop in the rates of mitochondrial ATP synthesis and F_1_-mediated ATP hydrolysis, and the absence of significant ATP-driven proton pumping across the mitochondrial membrane.

As was systematically observed with mutants with severe ATP synthase defects (25–27,39,44–48), the L_186_P strain had a somewhat high propensity to produce ρ^−^/ρ^0^ cells (>50% vs <5% for wild type yeast). Mutants with massive proton leaks through the F_O_ produce 100% ρ^−^/ρ^0^ cells because retention of functional mtDNA is then lethal by preventing the maintenance of a minimal electrochemical potential across the mitochondrial inner membrane (39). Indeed, without functional mtDNA the F_O_ cannot be synthesized and the mitochondrial membrane can be energized through the electrogenic exchange of glycolytic ATP against matrix-localized ADP combined to the hydrolysis of ATP by the F_1_ (these activities are controlled by nuclear genes). A lack of F_O_ activity also compromises (for as-yet-unknown reasons) the stability of the mitochondrial genome as was observed in strains lacking subunit *9*/*c* (25), subunit *6*/*a* (26) or one of the factors involved in the assembly of these proteins (47,48), which all produce at least 50% ρ^−^/ρ^0^ cells despite the absence of any F_O_-mediated proton leak. It is unlikely that the instability of L_186_P yeast results from F_O_-mediated protons leaks. Indeed, glucose cultures of this mutant contained a significant fraction (up to 50%) of ρ^+^ viable cells and despite a low electron transfer activity its mitochondria were able to sustain a significant and stable membrane potential when fed with electrons from ethanol (Fig. 4). Furthermore, consistent with previous work showing that the activity of F_O_ rather than the rate of F_1_-mediated ATP synthesis controls the rate of Complex IV biogenesis (41), this complex was down regulated in L_186_P yeast (Fig. 3). Further evidence for the existence of tight connections between the biogenesis of Complexes IV and V was recently provided by the detection of assembly intermediates containing subunits of both complexes, possibly as a mean to adjust their relative abundancy (42). Taking together, these observations indicate that the increased propensity of L_186_P yeast to produce ρ^−^/ρ^0^ cells is due to a lack of F_O_ activity.

**Figure 5:**
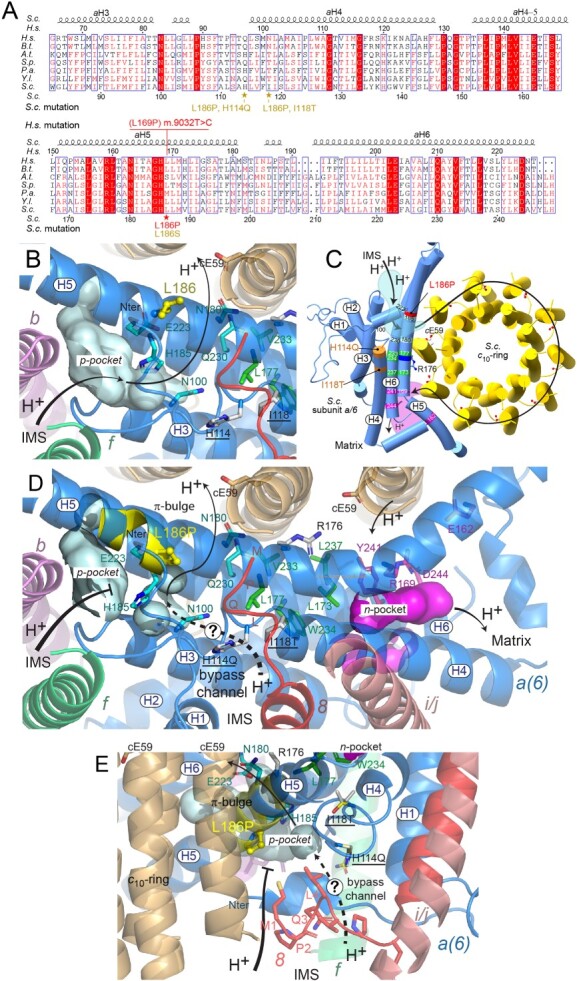
Evolutionary conservation and topology of the mutated subunit *a* residues. (**A**) Sequence alignment of helices *a*H3 to *a*H6 of subunit *a* from various species. The shown sequences are from *Homo sapiens* (*H.s.*), *Bos taurus* (*B.t.*), *Arabidopsis thaliana* (*A.t.*), *Schizosaccharomyces pombe* (*S.p.*), *Podospora anserina (P.a.), Yarrowia lipolytica* (*Y.l.*) and *Saccharomyces cerevisiae* (*S.c.*). Amino acid numbering in *H.s.* and *S.c.* subunits *a* is indicated above and below the alignments, respectively. As indicated in red, the m.9032T>C results in the replacement with proline of the leucine residue present at position 169 of *H.s*. subunit *a*. The equivalent mutation in yeast is L_186_P. The positions and amino acid changes induced by suppressors of L_186_P are indicated in yellow/brown. (**B**) Detail view of the *p*-pocket. Residues H_185_, E_223_, N_100_, N_180_ and Q_230_ (drawn as sticks with carbons colored in cyan) are important for moving protons from the external side of the inner membrane to the *c*-ring. L_186_ is drawn as yellow ball and stick. The H_114_ and I_118_ residues targeted by second-site suppressors are drawn as sticks with carbons colored in white. (**C**) Overall view from the intermembrane space (IMS) of the *a*/*c*_10_-ring in *S.c.* with the pathway (black line with arrows) along which protons are moved from the IMS to the mitochondrial matrix (19). The *p*- and *n*-pockets involved in this transfer are shown in cyan and purple background, respectively (as in panels **B** and **D**). The R_176_ residue of subunit *a* and the essential glutamate residue of subunit *c* (*c*E_59_) are essential for the activity of F_O_. (**D**) The π-bulge modification of *a*H5 induced by L_186_P is in yellow. Residues L_173_, L_177_, W_234_ and L_237_ (in green) belong the hydrophobic plug that separates the *p*- and *n*-site pockets. Subunits *b* (4), *f*, *8* and *i/j* are drawn as cartoons. For sake of clarity, the N-terminal helices of subunits *a* and *8* are drawn as loops. The dotted line indicates a possible pathway for protons towards the *c*-ring that is induced by the second-site genetic suppressors (H_114_Q and I_118_T) of L_186_P. Residues (E_162_, R_169_, Y_241_ and D_244_) important for moving protons along the *n*-pocket are in purple. (**E**) Side view of the p-pocket. A π-bulge due to the L_186_P mutation (colored in yellow), prevents the entry of protons into the *p*-pocket. In mutants H_114_Q and I_118_T, a bypass path between the N-terminal of subunit *8* and the C-terminal extremity of *a*H4 (drawn as loops) possibly allows the protons to access the bottom of the *p*-pocket. Mutated residues H_114_Q and I_118_T are drawn as sticks with carbons colored in yellow.

The subunit *a* and a ring of identical subunits *c* (8 in humans, 10 in yeast) are responsible for the transport of protons across the membrane domain (F_O_) of ATP synthase (Fig. 5C). A hydrophilic pocket within the subunit *a* on the external side of the inner membrane (referred to as *p*-pocket) allows protons from the intermembrane space to access an essential acidic residue in subunit *c* (*c*E_59_ in yeast) near the middle of the membrane (13,19–21). After an almost complete rotation of the *c*-ring, the protons are released and transferred to the mitochondrial matrix through a second hydrophilic pocket within the subunit *a* (*n*-pocket). The two pockets are separated by a plug of hydrophobic residues in subunit *a* near the middle of the membrane and close to it, in front of *c*E_59_, is a positively charged arginine residue belonging to subunit *a* (R_176_ in yeast) that is essential for F_O_ activity.

The amino acid change induced by the pathogenic m.9032T>C mutation (L_186P_ in yeast) is proximal to the *p*-pocket (Fig. 5B). This pocket is surrounded by segments of three subunit *a* α-helices (*a*H3, *a*H5 and *a*H6), the second transmembrane helix of subunit *4* (or *b*), the C-terminal helix of subunit *f*, the N-terminal domains of subunits *a* and *8* and a plug of hydrophobic subunit *a* residues near the middle of the membrane (L_173_, L_177_, V_233_, W_234_ and L_237_) (Fig. 5B, C). Based on studies in *E. coli* (49–51), protons would enter this pocket with the help of H_185_ and E_223_ and next moved to the *c*-ring via N_100_, N_180_ and Q_230_. Being located on *a*H5, the L_186_P change induced by m.9032T> C may disturb the structure of the *p*-pocket and compromise its proton-conduction activity. Proline residues are indeed known for their propensity to kink α-helices or to induce a more local distortion referred to as a π-bulge (52,53). Bending *a*H5 would certainly compromise assembly/stability of subunit *a* and hence increase its susceptibility to degradation. Since this was not observed, a π-bulge modification is more likely. As a result of this, the topology of H_185_ is changed and its distance with E_223_ increases, which could be the reason for the observed loss of F_O_-mediated proton transfer in the L_186_P mutant. The large recovery of ATP synthase function with a serine residue at position 186 is not very surprising considering the good ability of this type of residue to be incorporated within α-helices, thus allowing H_185_ to recover its normal topology. Being located 12 Å away from position 186, it is unlikely that the second-site suppressors H_114_Q and I_118_T can correct the π-bulge modification induced by L_186_P. The H_114_ residue interacts with a short helical segment at the N-termini of subunit *a* that caps the *p*-pocket and with a conserved motif (MPQL) at the beginning of subunit *8* that is supposed to stabilize the surface of subunit *a* in the membrane (54), while I_118_ is more deeply buried in the membrane beneath the MPQL motif close to the hydrophobic plug that separates the two proton conduction domains of subunit *a* (Fig. 5D). It is a reasonable assumption that H_114_Q and I_118_T enable the protons to regain access to the *c*-ring through a path that bypasses the inactive H_185_/E_223_ dyad. Possibly, the two second-site suppressor mutations allow the protons to be moved towards the bottom of the pocket from N-terminal domain of subunit *8* along the C-terminus of *a*H4 and are next moved by the triad of strictly conserved residues (N100, Q230 and N180) to the essential acidic residue of subunit *c* (E59 in yeast) (Fig. 5E). The low yield of ATP per electron transferred to oxygen observed with H_114_Q indicates that after their entry into the *p*-pocket the protons are channeled less efficiently towards the *c*-ring compared to wild type ATP synthase.

Intriguingly, Q and T are mostly present at the corresponding positions 114 and 118 in subunits *a* from other species, including humans. The ‘humanization’ of the yeast subunit *a* in response to a mutation with detrimental consequences is an interesting observation indicating that positions 114 and 118 (97 and 101 of the human subunit *a*) were possibly exploited during evolution to optimize F_O_ activity in those species with high energy demands and where the ATP is mostly produced in mitochondria. In this respect, it deserves to be highlighted that the ATP synthase of yeast is clearly less performing that the human enzyme, as evidenced by the need in yeast of more protons to make one ATP compared to humans (due to differences in *c*-ring stoichiometry) (55).

The present study demonstrates that the diseases induced by the leucine-to-proline change in subunit *a* induced by the m.9032T> C mutation is due to a block in F_O_-mediated transport between the external side of the inner membrane and the *c*-ring motor of ATP synthase. The possibility to bypass this mutation by second structural changes within the *p*-pocket is an interesting finding that opens a path for designing molecules that can improve oxidative phosphorylation in patients with mutations in the *p*-pocket.

## Materials and Methods

### Yeast strains and growth media

The sources and genotypes of the yeast strains used in this study are listed in Table 3. The media used for growing yeast strains were: YPGA (1% Bacto yeast extract, 1% Bacto Peptone, 2% or 10% glucose, 40 mg/L adenine), YPGalA (1% Bacto yeast extract, 1% Bacto Peptone, 2% galactose, 40 mg/L adenine), YPGlyA (1% Bacto yeast extract, 1% Bacto Peptone, 2% glycerol, 40 mg/L adenine). Media were solidified with 2% (w/v) Bacto agar. Growth curves were established with the Bioscreen CTM system.

**Table 3:** Genotypes and sources of yeast strains

Strain	Nuclear genotype	mtDNA	Source
DFS160	*MATα leu2Δ ura3–52 ade2–101 arg8::URA3 kar1–1*	ρ^0^	(56)
NB40-3C	*MATa lys2 leu2–3112 ura3–52 his3ΔHindIII arg8::hisG*	ρ^+^ *cox2–62*	(57)
MR6	*MAT**a** ade2–1 his3–11,15 trp1–1 leu2–3112 ura3–1 CAN1 arg8::HIS3*	ρ^+^	(26)
MR10	*MAT**a** ade2–1 his3–11,15 trp1–1 leu2–3112 ura3–1 CAN1 arg8::HIS3*	ρ^+^ *atp6::ARG8^m^*	(26)
SDC30	*MATα leu2Δ ura3–52 ade2–101 arg8::URA3 kar1–1*	ρ*^−^ ATP6 COX2*	(58)
EBY10a	*MATα leu2Δ ura3–52 ade2–101 arg8::URA3 kar1–1*	ρ*^−^ atp6-L186P*	This study
EBY10	*MAT**a** ade2–1 his3–11,15 trp1–1 leu2–3112 ura3–1 CAN1 arg8::HIS3*	ρ^+^ *atp6-L186P*	This study
EBY17	*MAT**a** ade2–1 his3–11,15 trp1–1 leu2–3112 ura3–1 CAN1 arg8::HIS3*	ρ^+^ *atp6-L186S*	This study
EBY18	*MAT**a** ade2–1 his3–11,15 trp1–1 leu2–3112 ura3–1 CAN1 arg8::HIS3*	ρ^+^ *atp6-L186P, H114Q*	This study
EBY20	*MAT**a** ade2–1 his3–11,15 trp1–1 leu2–3112 ura3–1 CAN1 arg8::HIS3*	ρ^+^ *atp6- L186P, I118T*	This study

### ATP6 mutagenesis

An equivalent of m.9032T>C (L_186_P) was introduced into a *Bam*HI-*Eco*RI fragment on the 5′ side of the yeast *ATP6* gene cloned into pUC19 (plasmid pSDC8, see (59)), using the Q5® Site-directed Mutagenesis Kit of NEB and primers 5’CACGACGTTGTAAAACGACGG**CCA**GTGAATTCACTATTGGTATCATTCAGGGATATGTCTG and 5’CAGACATATCCCTGAATGATACCAATAGTGAATTCAC**TGG**CCGTCGTTTTACAACGTCGTG (the mutagenic bases are in bold). The mutated *ATP6* fragment was cut with *Bam*HI and *Eco*RI and ligated at the same sites in plasmid pJM2 (56). This plasmid contains the yeast mitochondrial *COX2* gene as a genetic marker for mitochondrial transformation. The remaining part of *ATP6* was cut off from pSDC9 (45) with EcoRI and SapI and fused to the L_186_P fragment in pJM2. The resulting plasmid (pEB15) and the LEU2 plasmid Yep351 (60) were introduced into cells from ρ^0^ strain DFS160 using the biolistic PDS-1000/He particle delivery system (Bio-Rad), as described (11). Leu + clones with pEB15 in mitochondria (EBY10a) were identified by virtue of their capacity to restore respiratory competence in crosses with the ρ*^+^* strain NB40-3C in which the mitochondrial *COX2* gene is partially deleted (*cox2–62* mutation (57)). To introduce the L_186_P mutation in a complete (ρ^+^) mitochondrial genome, EBY10a was crossed with strain MR10 (26), which is derivative of wild type strain MR6 in which the coding sequence of *ATP6* is in-frame replaced with *ARG8^m^* (*atp6::ARG8^m^*). *ARG8^m^* is a mitochondrial version of a nuclear gene (*ARG8*) that encodes a protein involved in arginine biosynthesis (24). The EBY10a × MR10 crosses did not produce a single respiring clone, suggesting that the L_186_P mutation virtually abolishes ATP synthase function. ρ^+^ clones with the L_186_P mutation were therefore isolated based on their incapacity to grow in media lacking arginine (due to replacement of *atp6::ARG8^m^* with the mutated *ATP6* gene) and to recover respiratory competence after crossing with SDC30, which is a ρ^−^ synthetic strain containing in mitochondria only the *ATP6* and *COX2* genes (26). The presence in these clones (called EBY10) of the L_186_P mutation was verified by DNA sequencing with primers oATP6–1 5′TAATATACGGGGGTGGGTCCCTCAC and oATP6–10 5′GGGCCGAACTCCGAAGGAGTAAG. Due to the presence in EBY10a of the nuclear karyogamy delaying *kar1–1* mutation (61), the ρ^+^ recombinant clones could be isolated in the haploid nuclear background of wild type strain MR6 from which MR10 was derived (26).

### Mitochondrial DNA stability in L_186_P yeast

To evaluate their content in cells with large deletions in mitochondrial DNA (ρ^−^) or totally devoid of this DNA (ρ^0^), cultures of L_186_P yeast were plated for single colonies on glucose plates. About 200 of these colonies were replica crossed with cells from strain SDC30 on glucose plates. After one-night incubation, the mated cells were replicated on glycerol plates. The crosses that produced cells growing on glycerol originate from L_186_P subclones with a complete (ρ^+^) mitochondrial genome, while the ρ−/ρ^0^ cells present in the cultures of L_186_P yeast result in progenies entirely unable to grow on glycerol.

### Selection of revertants from L_186_P yeast

Three genetically independent L_186_P (EBY10) clones were grown for one night in rich glucose (YPGA). Cells were centrifuged and residual glucose removed by two washings with water. They were then spread on rich glycerol (YPGlyA) medium and incubated at 28°C for 21 days. Twelve revertants were picked up and genetically purified by subcloning on YPGlyA. The *ATP6* gene of these clones was PCR-amplified and sequenced entirely, which led to identification of 3 different intragenic suppressors (see below).

### Mitochondrial respiration, ATP synthesis/hydrolysis and membrane potential

Mitochondria were prepared from yeast cells grown in rich galactose (YPGalA) at 28°C by the method described in (62). Protein content in mitochondrial preparations was determined according to (63) in the presence of 5% SDS. For respiration and ATP synthesis assays, mitochondria were diluted to 0.075 mg/mL in respiration buffer (10 mM Tris-maleate (pH 6.8), 0.65 M sorbitol, 0.3 mM EGTA and 3 mM potassium phosphate). Oxygen consumption rates were measured using a Clarke electrode after adding consecutively 4 mM NADH (state 4 respiration), 150 μM ADP (state 3) and 4 μM carbonyl cyanide m-chlorophenylhydrazone (CCCP) (uncoupled respiration), as previously described (64). The rates of ATP synthesis in mitochondria respiring from NADH were determined in the presence of externally added 750 μM ADP, in the absence and presence of oligomycin (3 μg/mL), taking aliquots every 30 seconds and stopping the reaction with 3.5% (w/v) perchloric acid, 12.5 mM EDTA. The amounts of ATP in the samples were quantified using the Kinase-Glo Max Luminescence Kinase Assay (Promega) and a Beckman Coulter’s Paradigm Plate Reader. Variations in transmembrane potential (ΔΨ) were evaluated in the respiration buffer containing 0.15 mg/mL of mitochondrial proteins and 0.5 μg/mL of Rhodamine 123 (λ_exc_ of 485 nm and λ_emi_ of 533 nm) under constant stirring using a Cary Eclipse Fluorescence Spectrophotometer (Agilent Technologies, Santa Clara, CA, USA), in the presence of 75 μM ADP, 10 μL/mL ethanol, 2 mM potassium cyanide, 4 μg/mL oligomycin and 4 μM CCCP, as described in (37). The specific ATPase activity at pH 8.4 in non-osmotically protected mitochondria was measured in the absence and presence of oligomycin (3 μg/mL), as described in (65).

### BN- and SDS-PAGE analyses

Blue native-PAGE experiments were carried out as described (38) with 200 μg of mitochondrial proteins in 100 μL of extraction buffer (30 mM HEPES pH 6.8, 150 mM potassium acetate, 12% glycerol, 2 mM 6-aminocaproic acid, 1 mM EGTA, 1.5% digitonin (Sigma)), supplemented with one protease inhibitor cocktail tablet (Roche) per 10 mL and 1 mM PMSF. After 30 minutes incubation on ice, the samples were cleared by centrifugation (14 000 rpm, 4°C, 30 min), supplemented with 4.5 μL of loading dye (5% Serva Blue G-250, 750 mM 6-aminocaproic acid), run on NativePAGE^TM^ 3–12% Bis-Tris Gels (Invitrogen) and finally transferred onto PVDF for protein detection by Western blot with polyclonal antibodies against Atp1 (a kind gift from Marie-France Giraud, Bordeaux) at 1:10000 dilution and peroxidase-labeled antibodies (Promega) at a 1:5000 dilution and the ECL reagent of Pierce™ ECL Western Blotting Substrate (ThermoScientific). SDS-PAGE analysis and immunological detection of mitochondrial proteins was performed as previously described (66), with antibodies against ATP synthase subunits Atp2, Atp6, Atp7, the Cox2 subunit of Complex IV and porin (Por1) for normalization (the Atp2, Atp6 and Atp7 antibodies were provided by J. Velours; those against Cox2 and porin were purchased from Molecular Probes). The open source program ImageJ (http://rsbweb.nih.gov/ij/) was used to quantify the relevant immunological signals.

### Amino-acid alignments and topology of subunit *a* mutations

Multiple sequences of ATP synthase subunits *a* of various origins were aligned and drawn using Clustal Omega (67) and Espript 3.0 (68), respectively. Molecular views of subunit *a* and *c*_10_-ring were obtained from the dimeric F_o_ domain of *S. cerevisiae* ATP synthase (pdb_id: 6b8h, (19)). The shown structures were drawn using ChimeraX (69) and PyMOL Molecular Graphic System (70).

### Statistical analysis

At least three biological and three technical replicates were performed for all reported experiments. The *t*-test was used for all data sets. Significance and confidence level were set at *P*-0.05.

*Conflict of Interest statement*. The authors declare that they have no competing or financial interests.

## Funding

This work was supported by a grant from the National Science Center of Poland [2016/23/B/NZ3/02098] to R.K. and Association Française contre les Myopathies [AFM #22382] to D.T.T.

## Author Contributions

E.B. constructed plasmids and strains; C.P., E.B. and K.N. isolated mitochondria and analyzed their properties; A.D. and C.C. performed the structural modeling analyses; D.T.T., J.PdR. and R.K. wrote the manuscript and designed the work.

## Statement of Ethics

The permission number for work with genetically modified microorganisms (GMM I) for RK is 01.2–28/201.
